# Results from a Patient-Based Health Education Intervention in Reducing Antibiotic Use for Acute Upper Respiratory Tract Infections in the Private Sector Primary Care Setting in Singapore

**DOI:** 10.1128/AAC.02257-16

**Published:** 2017-04-24

**Authors:** Magdalene Hui Min Lee, Darius Shaw Teng Pan, Joyce Huixin Huang, Mark I-Cheng Chen, Joash Wen Chen Chong, Ee Hui Goh, Lili Jiang, Yee Sin Leo, Tau Hong Lee, Chia Siong Wong, Victor Weng Keong Loh, Fong Seng Lim, Adrian Zhongxian Poh, Tat Yean Tham, Wei Mon Wong, Yue Yu

**Affiliations:** a Yong Loo Lin School of Medicine, National University of Singapore, Singapore; b Saw Swee Hock School of Public Health, National University of Singapore, Singapore; c Institute of Infectious Diseases and Epidemiology, Communicable Disease Centre, Tan Tock Seng Hospital, Singapore; d Division of Family Medicine, Department of Medicine, University Medicine Cluster, National University Hospital System, Singapore; e Lee Kong Chian School of Medicine, Nan Yang Technological University, Singapore; f Frontier Healthcare Group, Singapore; g Division of Primary Care, Raffles Medical Group, Singapore; h Duke NUS Graduate Medical School, National University of Singapore, Singapore

**Keywords:** upper respiratory tract infection, intervention, antibiotics, antimicrobial stewardship

## Abstract

We investigated the efficacy of patient-targeted education in reducing antibiotic prescriptions for upper respiratory tract infections (URTIs) among adults in the private primary care setting in Singapore. Our randomized controlled trial enrolled patients aged 21 years and above presenting at general practitioner (GP) clinics with URTI symptoms for 7 days or less. Intervention arm patients were verbally educated via pamphlets about the etiology of URTIs, the role of antibiotics in treating URTIs, and the consequences of inappropriate antibiotic use. Control arm patients were educated on influenza vaccinations. Both arms were compared regarding the proportions prescribed antibiotics and the patients' postconsultation views. A total of 914 patients consulting 35 doctors from 24 clinics completed the study (457 in each arm). The demographics of patients in both arms were similar, and 19.1% were prescribed an antibiotic, but this varied from 0% to 70% for individual GPs. The intervention did not significantly reduce antibiotic prescriptions (odds ratio [OR], 1.20; 95% confidence interval [CI], 0.83–1.73) except in patients of Indian ethnicity (OR, 0.28; 95% CI, 0.09–0.93). Positive associations between the intervention and the view that antibiotics were not needed most of the time for URTIs (*P* = 0.047) and on being worried about the side effects of antibiotics (*P* = 0.018) were restricted to the Indian subgroup. GPs in limited liability partnerships or clinic chains prescribed less (OR, 0.36; 95% CI, 0.14 to 0.92), while certain inappropriate patient responses were associated with the receipt of antibiotics. Follow-up studies to investigate differences in responses to educational programs between ethnicities and to explore GP-targeted interventions are recommended.

## INTRODUCTION

Antibiotic resistance is a global public health concern (1). Resistant bacteria are associated with greater morbidity, mortality, and socioeconomic costs (1, 2), with the development of resistance linked to the overuse of antibiotics (2–4). Upper respiratory tract infections (URTIs) are the most common condition seen in primary care settings in Singapore (5) and may be a major source of antibiotic overuse (6). Antibiotic prescriptions for URTIs in primary care settings also remain high in other parts of the world (3, 7, 8). This is in spite of the current evidence, which does not support antibiotic use in most cases of URTIs (9, 10), since URTIs are frequently of a viral etiology, are often self-limiting, and seldom lead to serious complications (11, 12). Moreover, the overuse of antibiotics for URTIs promotes the selection of antibiotic-resistant bacteria (13), increases the risk of adverse drug reactions (14), and increases costs (1, 2). The factors driving inappropriate antibiotic prescribing for URTIs are multifactorial and include the patients' inadequate knowledge on appropriate antibiotic use for URTIs (6, 15) and direct or indirect pressure from patients on physicians to prescribe antibiotics (16, 17).

Reducing antibiotic prescriptions can decrease the prevalence of antibiotic-resistant bacteria (18). Interventions to reduce antibiotic prescriptions for URTIs have been attempted in the United States and Europe, with mixed results (19). While there are no published studies on such interventions in Singapore, local studies have found that many patients seeing primary care physicians had several misconceptions regarding antibiotic use in URTI, including that antibiotics are effective against viruses, cure URTIs faster, and are necessary for URTIs (6, 20). Those who knew that URTIs are self-resolving had more appropriate health-seeking behaviors (6). Hence, we hypothesized that patients' misconceptions were a strong factor for antibiotic overuse in Singapore, and that correcting these misconceptions through patient education would reduce antibiotic prescriptions.

We designed a patient-targeted intervention via educational pamphlets and verbal counseling on the causes of URTIs and the role of antibiotics in treating URTIs. We performed a randomized controlled trial to investigate its efficacy in the private general practitioner (GP) setting, which accounts for 87% of URTI consults in Singapore (5). Our primary aim was to assess the efficacy of our intervention in reducing antibiotic prescriptions, and our secondary aim was to assess patients' postintervention views about the use of antibiotics for URTIs. Finally, we also present other patient and GP factors found to be associated with receiving an antibiotic.

(This work was presented in part as a poster presentation at the 11th Student Medical-Nursing Education Conference, 15 August 2015, as an oral presentation at the 1st International Meeting on Respiratory Pathogens, 2 to 4 September 2015, and as a poster presentation at the Singapore Health and Biomedical Congress, 2 to 3 October 2015.)

## RESULTS

**FIG 1:**
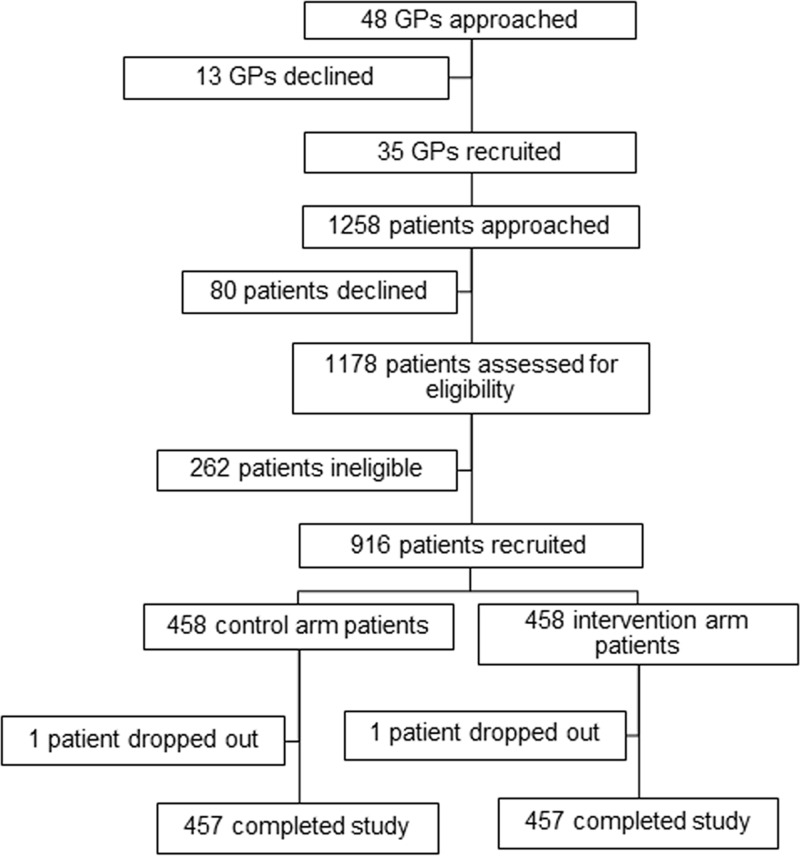
Recruitment of participating GPs and patients.

Of the 48 GPs approached, 35 agreed to participate in the study (Fig. 1). A total of 1,258 of their patients were approached; 80 declined and 262 were ineligible. The remaining 916 patients were randomized into control and intervention arms. Two patients dropped out, with 457 patients from each arm completing the study.

The median age of participating patients was 35 years (interquartile range [IQR], 28 to 45; Table 1). Among these, 48.8% were male, 68.9% were Chinese, and 74.4% had postsecondary qualifications or higher. Distributions for age, sex, ethnicity, and educational qualifications were similar between both arms.

**TABLE 1:** Patients' demographic characteristics in control and intervention arms

Characteristic	Patient arms		
	Total (*n* = 914)	Control (*n* = 457)	Intervention (*n*= 457)
Age (yr) (median [IQR])^*a*^	35 (28–45)	35 (28–45)	36 (28–44)
Male sex (*n* [%])	454 (48.8)	218 (47.0)	236 (50.5)
Ethnicity (*n* [%])			
Chinese	630 (68.9)	310 (67.8)	320 (70.0)
Malay	116 (12.7)	62 (13.6)	54 (11.8)
Indian	99 (10.8)	46 (10.1)	53 (11.6)
Others	69 (7.6)	39 (8.5)	30 (6.6)
Highest qualification attained (*n* [%])^*b*^			
Secondary and below	234 (25.6)	118 (25.8)	116 (25.4)
Postsecondary and above	679 (74.4)	339 (74.5)	340 (74.4)

a IQR, interquartile range.

b Missing data from one patient in the intervention arm.

Overall, the intervention did not significantly affect antibiotic prescriptions (Table 2), with 94 (20.6%) intervention and 81 (17.7%) control arm patients receiving prescriptions for antibiotics (*P* = 0.313). However, in the Indian ethnic subgroup, the intervention arm patients received significantly fewer antibiotic prescriptions (odds ratio [OR], 0.28; 95% confidence interval [CI], 0.09 to 0.93; *P* = 0.037 on the stratified analysis), whereas there was no significant difference among those of other ethnicities; similar results were obtained after a multivariate analysis adjusting for potential confounding by the factors investigated in Table 3. However, no significant differences in prescriptions between intervention and control arms were revealed on stratification by patient age, sex, or educational qualifications (data not shown).

**TABLE 2:** Overall intervention effect on prescriptions and subgroup analysis by ethnicity

Subgroup	Control arm		Intervention arm		Univariate/stratified analysis		Multivariate analysis^*a*^	
	Total no. of patients	No. of patients (%) receiving antibiotics	Total no. of patients	No. of patients (%) receiving antibiotics	OR (95% CI)^*b*^	*P* value	OR (95% CI)^*b*^	*P* value
All patients	457	81 (17.7)	457	94 (20.6)	1.20 (0.83–1.73)	0.322		
Ethnicity								
Chinese	310	52 (16.8)	320	72 (22.5)	1.48 (0.96–2.31)	0.075	1.46 (0.91–2.35)	0.113
Malay	62	11 (17.7)	54	9 (16.7)	0.78 (0.27–2.23)	0.652	0.56 (0.19–1.69)	0.307
Indians	46	13 (28.3)	53	6 (11.3)	0.28 (0.09–0.93)	0.037	0.27 (0.08–0.97)	0.044
Others	39	5 (12.8)	30	7 (23.3)	2.25 (0.56–9.07)	0.251	2.07 (0.46–9.31)	0.339

a Multivariate analysis including all factors from Table 3.

b With control arm as reference category. OR, odds ratio; CI, confidence interval.

**TABLE 3:** Associations of patient and doctor-level factors with receiving an antibiotic

Factor	Univariate analysis		Multivariate analysis	
	OR (95% CI)^*a*^	*P* value	OR (95% CI)	*P* value
Age group (vs those aged 21–34 years)				
35–49	1.52 (1.00–2.32)	0.052	1.55 (0.97–2.50)	0.068
50–64	1.96 (1.13–3.39)	0.016	2.43 (1.27–4.67)	0.008
≥65	2.66 (1.22–5.8)	0.014	3.71 (1.42–9.72)	0.008
Sex (vs male)				
Female	0.87 (0.60–1.25)	0.447	0.89 (0.59–1.33)	0.556
Ethnicity (vs Chinese)				
Malay	0.97 (0.55–1.71)	0.918	1.55 (0.67–3.60)	0.308
Indian	1.11 (0.61–2.02)	0.742	2.13 (0.87–5.23)	0.099
Others	0.73 (0.35–1.49)	0.386	0.58 (0.17–1.93)	0.372
Highest qualification (vs primary)				
Secondary	0.62 (0.28–1.39)	0.243	0.77 (0.30–1.97)	0.590
Above secondary	0.59 (0.28–1.23)	0.157	1.14 (0.44–2.94)	0.793
Patient knowledge and beliefs (vs appropriate response)				
I want to receive antibiotics^*b*^	3.17 (2.14–4.70)	<0.001	3.16 (2.04–4.89)	<0.001
Antibiotics cure respiratory infections faster^*b*^	2.02 (1.31–3.12)	0.001	1.57 (0.96–2.56)	0.071
Antibiotics are effective against viral infections^*c*^	1.30 (0.82–2.06)	0.271	1.24 (0.73–2.10)	0.433
There are no side effects from the consumption of antibiotics^*c*^	0.71 (0.49–1.04)	0.078	0.65 (0.43–0.99)	0.046
Viruses cause respiratory infections^*d*^	1.30 (0.89–1.90)	0.174	1.58 (1.03–2.41)	0.036
URTI^*e*^ resolves on its own^*d*^	1.48 (1.02–2.15)	0.040	1.39 (0.92–2.09)	0.115
Antibiotic-resistant infections are not easily killed by antibiotics^*d*^	0.83 (0.57–1.20)	0.311	0.79 (0.52–1.20)	0.270
Consuming too much antibiotics decreases the effectiveness of the antibiotics^*d*^	0.71 (0.44–1.15)	0.163	0.61 (0.36–1.05)	0.075
GP characteristics				
Female (vs male)	1.25 (0.47–3.33)	0.661	1.26 (0.48–3.36)	0.640
Number of years qualified^*f*^	1.01 (0.97–1.06)	0.699	0.99 (0.94–1.05)	0.819
Being involved a limited-liability partnership or clinic chain	0.32 (0.14–0.73)	0.006	0.36 (0.14–0.92)	0.033

a CI, confidence interval, OR, odds ratio.

b Strongly agree/agree as inappropriate response.

c Yes/do not know as inappropriate response.

d No/do not know as inappropriate response.

e URTI, upper respiratory tract infection.

f Refers to the number of years after the GP qualified as a medical practitioner.

**FIG 2:**
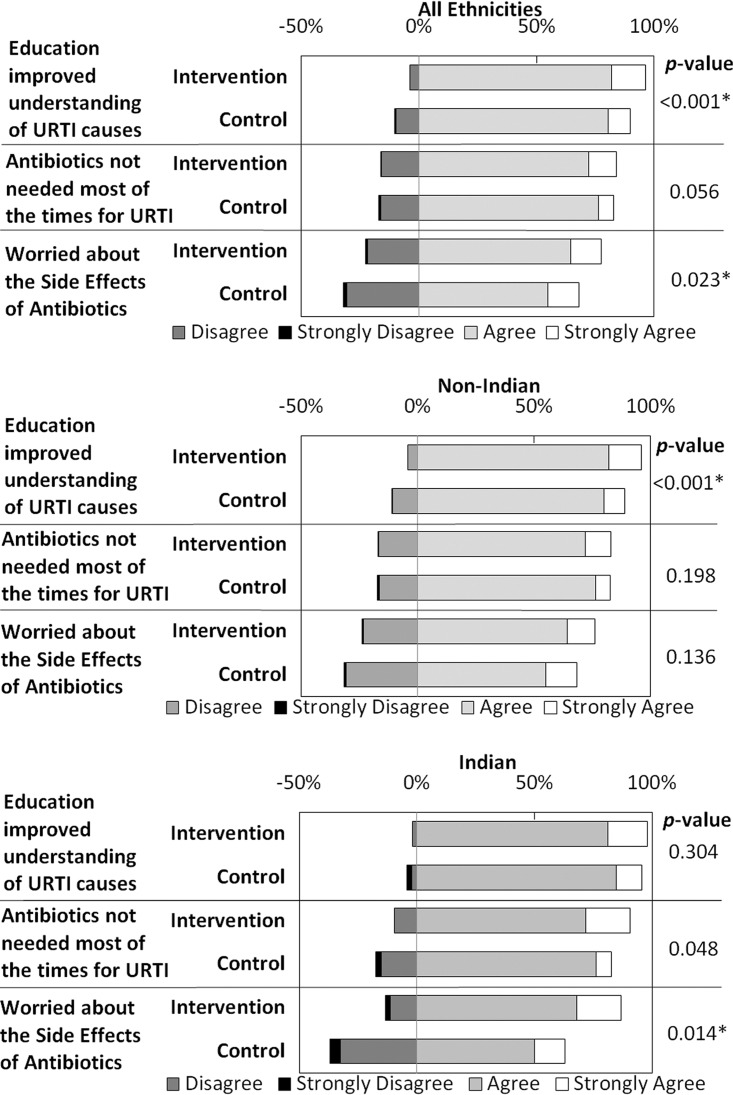
Effects of intervention on patients' views about URTI and antibiotic use. Black, dark gray, light gray, and white sections of the bars represent strongly disagree, disagree, agree, and strongly agree, respectively, with *P* values from Mann-Whitney U tests given for the comparison of responses between the control and intervention arms.

Figure 2 compares patients' postconsultation views of URTIs and the antibiotic use by study arm. Overall, there was a significant positive association between the intervention and agreement with the statement that the education improved the patient's understanding of URTI causes (*P* < 0.001). However, significant positive effects on views about antibiotics were restricted to those of Indian ethnicity, both on agreement that antibiotics are not needed most of the time for URTIs (*P* = 0.048) and on being worried about the side effects of antibiotics (*P* = 0.014).

**FIG 3:**
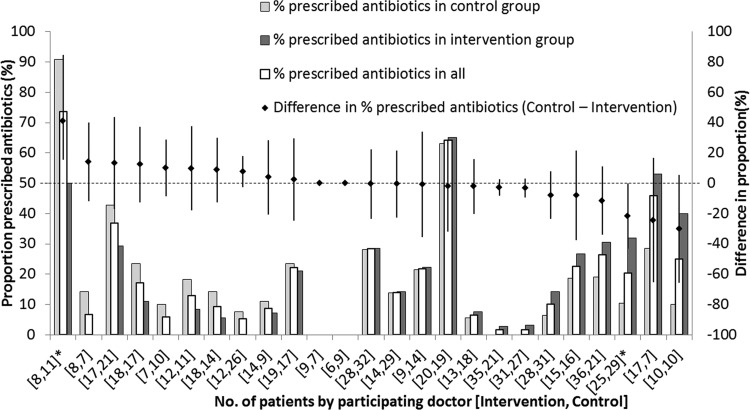
Proportion of patients that were prescribed antibiotics in intervention and control arms, stratified by participating GP. Bars (left vertical axis) represent the percentages of control patients (dark gray), all patients (white with bold outline), and intervention patients (light gray) that were prescribed antibiotics. The diamonds coinciding with the middle bars are the differences in proportion with antibiotic prescriptions between the two arms (control − intervention, with error bars denoting the 95% confidence intervals [right vertical axis]). The numbers of interventions and control arm patients for that GP are in the brackets below the bars; two of the GPs are starred because the difference was statistically significant.

Among GPs who contributed more than 10 patients, the proportion that were prescribed antibiotics by each GP varied widely from 0 to 70%. Ten GPs were more likely to prescribe antibiotics for control than intervention arm patients; the converse was true for 13 GPs, and the difference was statistically significant for only one GP each way (Fig. 3). A stratified analysis did not reveal associations between any characteristics of participating GPs and the efficacy of the intervention.

Table 3 highlights the nonintervention-related patient and doctor-level factors associated with receiving an antibiotic. Compared with those aged 21 to 34 years, those in older age groups were significantly more likely to receive an antibiotic in both univariate and multivariate analyses, with those aged ≥65 years having the highest odds of receiving an antibiotic (OR, 3.71; 95% CI, 1.42 to 9.72; *P* = 0.008 on multivariate analysis). Patients who wanted to receive antibiotics were more likely to receive antibiotics on both univariate and multivariate (OR, 3.16; 95% CI, 2.04 to 4.89; *P* < 0.001) analyses. Inappropriate responses to some questions also significantly increased the odds of the participant receiving an antibiotic on univariate (“Antibiotics cure respiratory infections faster,” OR, 2.02; 95% CI, 1.31 to 3.12; *P* = 0.001; “URTI resolves on its own,” OR, 1.48; 95% CI, 1.02 to 2.15; *P* = 0.040) and multivariate (“Viruses cause respiratory infections,” OR, 1.58; 95% CI, 1.03 to 2.41; *P* = 0.036) analyses. Giving an inappropriate answer to “There are no side effects from the consumption of antibiotics” was associated with decreased antibiotic prescriptions on univariate analysis, which became statistically significant in the multivariate analysis (OR, 0.65; 95% CI, 0.43 to 0.99; *P* = 0.046), suggesting that those who received antibiotics were actually more likely to know (in the preconsultation assessment) that antibiotics had side effects. Among the doctor-level characteristics, being involved with a limited-liability partnership or clinic chain was significantly associated with fewer antibiotic prescriptions on both univariate and multivariate (OR, 0.36; 95% CI, 0.14 to 0.92; *P* = 0.033) analyses.

## DISCUSSION

Patients who received our educational intervention reported that it improved their understanding of URTI causes. However, this did not reduce antibiotic prescription rates except in patients of Indian ethnicity, among whom the intervention also had a demonstrable association with an increased awareness that antibiotics are not indicated most of the time for URTIs and of the potential side effects of antibiotics. What we did observe was that across the 35 GPs, the proportion of patients for whom antibiotics were prescribed varied widely. Moreover, the finding that GPs who were part of a bigger corporate entity, such as a limited-liability partnership or clinic chain, were less likely to prescribe antibiotics suggests that a possible direction for future interventions might involve measures targeting the GPs.

Previous trials of patient information leaflets have had mixed results, with most reporting either significant reductions in antibiotic prescription and use or nonsignificant reductions (21). It is possible that the overall intervention effect was more modest than the 40% relative reduction in prescriptions, which our sample size would have been adequately powered to detect (given the overall prescription rate we found of about 19%). There are also other possible explanations. First, it has been postulated that patients undergo a complex process of behavioral change, as exemplified by the transtheoretical model (22), with time needed for an educational intervention to demonstrate its effects. If so, prescriptions at subsequent consultations might be more suitable for assessing the efficacy of such interventions (23) than our current study design, which focused on prescriptions in the same visit. Second, there are multiple links in the causal chain between patient perceptions and eventual antibiotic prescription, as well as other factors that influence antibiotic prescriptions. In our non-Indian population, although the intervention resulted in more patients agreeing that their understanding of URTI causes had improved, it did not affect antibiotic prescriptions. A previous trial similarly found that an educational intervention for parents modified parental attitudes about the use of antibiotics but did not significantly reduce antibiotic prescriptions for their children (24). Also, our health education was designed to target inappropriate beliefs about URTI and antibiotics identified in a previous local study (6); in our patient population, inadequate knowledge was also highly prevalent, but not as pervasive. For instance, while only 36% from the previous study knew that “URTI resolves on its own,” a much higher proportion of 51% gave the appropriate answer among our patients (20). This would have reduced the pool of patients benefiting from a health education intervention targeting inaccurate knowledge. Moreover, as we only targeted patients' perceptions, other factors influencing antibiotic prescriptions might have been active.

Furthermore, there may be ethnic variations in the response to educational interventions among the patient population in Singapore, as suggested by how the positive effects of the intervention were restricted to patients of Indian ethnicity. These findings might be explained by a differing receptiveness to health education. One local study found that, among Chinese, Malays, and Indians, which are the three major ethnic groups in Singapore, Malays and Indians were more willing to participate in health education programs than the Chinese (25). Various degrees of language proficiency may also have played a role. One important limitation we faced was that our educational pamphlet was designed only in English. Moreover, due to the language competencies of our field researchers, verbal counseling was provided only in English or Mandarin; hence, we selected only patients conversant in these two languages. However, the degree to which someone was competent in the language they were counseled in may have varied by the ethnicity. For instance, local census data show that only 21.2% of Malays who are literate in English speak English at home, compared with 49.8% of Indians and 45.2% of Chinese (26). Therefore, ethnic differences in the extent to which patients understood the education we provided may have arisen. Ethnic and cultural factors have also been noted to affect health education outcomes in other countries (27). We suggest that these factors be investigated further, perhaps through qualitative research methods, and accounted for in future health education interventions. For now, what we found was that patients who wanted antibiotics indeed had a higher likelihood of receiving an antibiotic, which is consistent with prior literature in other settings (28). Inappropriate responses to questions such as “Antibiotics cure respiratory infections faster,” “URTI resolves on its own,” and “Viruses cause respiratory infections” were also found to be associated with antibiotic prescription, either on univariate or multivariate analyses; we had previously shown that some of these are associated with wanting antibiotics (20). Further understanding of how inaccurate knowledge might act independently or via a patient's expectations for an antibiotic may require more sophisticated methods, such as latent variable analyses and structural equation models. On the other hand, we found, unexpectedly, that those who gave an appropriate answer to “There are no side effects from the consumption of antibiotics” had higher odds of receiving an antibiotic than those who gave the inappropriate response; we hypothesize that some who gave the appropriate response may have previously asked for antibiotics and thus already received prior counseling on potential side effects.

Finally, although not the original objective of our study, it is worth highlighting the high overall prescriptions of 19.1% compared with those from other Asian countries with similar health care standards, such as Taiwan (6%) (29) and Hong Kong (5%) (30), and even more importantly, the wide variation in antibiotic prescriptions from 0 to 70% across GPs, which suggests that GP factors, such as clinical judgment and prescribing habits (31, 32), and health care system factors, such as health policies (33), were more critical in determining whether an antibiotic was prescribed than what could be achieved through our patient-targeted intervention. In other studies on antibiotics prescriptions, it was also highlighted that recipient characteristics did not alter the likelihood that an antibiotic was prescribed, but rather the provider was a key factor affecting prescriptions (34, 35). In addition, one study found that being in a limited-liability partnership or group practice was a factor associated with decreased average prescription rates (36). Another study found that public sector clinics prescribed less antibiotics than private sector clinics (37). Similarly, our study also noted that being involved in a larger corporate entity, such as a limited-liability partnership or clinic chain practice, was associated with decreased antibiotic prescriptions for URTI. Possible explanations for this may be that larger groups may have established guidelines for antibiotic stewardship, as well as a potential peer effect, with the GPs in such groups aware if they are prescribing more than their colleagues. Overall, these findings point to room for optimizing antibiotic prescription practices in Singapore in private sector clinics. More research is needed on GP-level factors that influence prescriptions and potential interventions for reducing antibiotic prescriptions that are relevant to the context of private sector GPs. For instance, deferred antibiotic prescription, found to be effective elsewhere (38), has anecdotally been practiced by some GPs (39). However, because antibiotics in Singapore are generally prescribed by a dispensary within the clinics themselves rather than a local pharmacy, a deferred prescription would entail a return visit to the clinic, and can potentially be viewed as an inconvenience or source of additional costs.

Our study had several limitations. The number of GPs was relatively small and not representative of GPs across Singapore, since many participating GPs were from an academic medicine network and thus might be more likely to value prescription habits in accordance with the principles of antimicrobial stewardship. Although approaching GPs randomly sampled from a national register might have yielded a more accurate representation, this was not attempted as our previous study using such a method resulted in a dismal response rate (40). Second, having GPs as part of the study may inadvertently influence their prescribing practice, as the knowledge that their practices are being observed may make them less likely to prescribe an antibiotic than in their usual practice (41). Third, disproportionate numbers of patients per GP, from 6 to 60, were recruited, which was due to variations in the numbers of patients visiting each GP during the 2-week study period. Fourth, it would have been ideal to educate patients more thoroughly over a longer duration, but as explained previously, this was not possible due to the GP clinic setting. Related to this, while our intervention was meant to approximate what would be a realistic busy clinic setting where a pamphlet is given and counseling is done by someone other than the attending physician (such as the clinic receptionist), patients are likely to not have trusted our medical students as much as they would have trusted the regular clinic staff, and this may have compromised the effect of the intervention. Sixth, our study intervened at the point of care, as we sampled patients suffering from URTIs who were already attending a clinic. This may not have given sufficient time for the education to take effect, especially for patients who were already intending to see the GP for antibiotics. Finally, as alluded to earlier, the actual use of the prescribed antibiotics and longer term effects on future GP consultations might have been better outcome measures for assessing the true effect of our intervention.

### Conclusions.

Our study found that an intervention for reducing antibiotic prescriptions in Singapore's primary health care setting may only have been effective in a small subset of patients. Although patients in the intervention arm reported an improved understanding of URTI causes, the intervention was associated with reduced antibiotic prescriptions and increased awareness about the appropriate use of antibiotics for URTIs and the side effects of antibiotics in the Indian ethnic subgroup only, and follow-up studies to investigate differences in responses to educational programs between ethnic groups might facilitate the design of more targeted patient-level interventions. These might potentially focus on the misconceptions and inappropriate attitudes we identified to be associated with receiving an antibiotic. On the other hand, the wide variation in antibiotic prescriptions across GPs and our finding that being part of a larger corporate entity was associated with lower rates for prescribing antibiotics suggests that health system factors have a substantial influence and that future directions for improving antimicrobial stewardship may lie in GP-targeted interventions.

## MATERIALS AND METHODS

### Study design and setting.

We conducted a two-arm, parallel group randomized controlled trial over nine working days (from 9 to 23 February 2015). GPs from the academic medicine network affiliated with the National University of Singapore (NUS) and three major GP clinic chains were approached via email. We then visited the GPs who responded to assess their clinics' suitability for the study, to explain the study objectives and execution, to address their concerns, and to obtain their consent. In all, 35 GPs were recruited from 24 clinics of various sizes, including solo and group practices in residential and commercial areas across Singapore.

A total of 38 fourth-year medical students from NUS Yong Loo Lin School of Medicine (YLLSoM) were deployed in pairs as field researchers to participating clinics during operating hours. These students had undergone a full-day training program that included video demonstrations, simulations, and role-play, which familiarized all researchers with the study protocol, standardized questionnaire administration, and health education delivery.

The inclusion criteria were patients aged 21 years and older and presenting with at least one of four URTI symptoms (runny nose, blocked nose, cough, or sore throat) for 7 days or less at participating clinics. Patients were excluded if they had previously sought medical consultation for the same symptoms, were on long-term immunosuppressive or oral corticosteroid medications, had chronic kidney disease, had a history of advanced stage or metastatic cancer, or were not conversant in English or Mandarin. Eligible patients who provided written consent were enrolled in the study.

Following enrollment, the first researcher administered an interviewer-assisted preconsultation questionnaire on the patient's demographic characteristics and details of the presenting illness. Each patient was then randomly allocated to either the control or intervention arm using sequential envelopes containing computer-generated assignments based on simple block randomization. We chose to randomize at the patient level rather than the cluster level to avoid the confounding effect of variations in GPs' antibiotic prescription practices. Although patient level randomization carried the risk of contamination, the researchers observed that this risk was low as patients generally did not communicate with each other.

Patients in the intervention arm were educated on causes of URTIs, the role of antibiotics in treating URTIs, and the consequences of inappropriate antibiotic use. The education pamphlets and scripts used can be found in Text S1 in the supplemental material. Patients in the control arm were educated on influenza and influenza vaccinations, a topic relevant to the patients' presenting complaints but not expected to directly influence antibiotic prescription. In both arms, this took the form of verbal counseling by the first researcher for about 3 min using standardized scripts in English or Mandarin with reference to the educational pamphlets provided. The pamphlets used were in English only. Patients were allowed to ask questions during this time, and all questions were answered. We chose to counsel patients for 3 min as this was sufficient time to impart basic knowledge and address fundamental misconceptions. A longer period of counseling might have adversely affected clinic flow and business, and resulted in a higher patient decline or drop-out rate.

After the patient's consultation with the GP, the second researcher, who was not involved in the preconsultation questionnaire or education, administered an interviewer-assisted postconsultation questionnaire on the patient's views about URTI causes and antibiotic use on a 4-point Likert scale and noted the medications prescribed.

### Health education materials.

The intervention pamphlets and counseling scripts were designed on the basis of information from patient information pamphlets and booklets from the Health Promotion Board (HPB), Singapore (42, 43) and the Centers for Disease Control and Prevention (CDC), United States (44). Several primary care and infectious disease physicians and public health experts were also consulted. The education material for the intervention group specifically addressed key misconceptions identified in the previous study of patients with URTIs at local primary care clinics (6). It was first tested on layperson volunteers, then field-tested during a pilot study in December 2014 and refined on the basis of feedback from participating GPs and patients.

### Blinding.

Attempts were made keep the GPs and researchers assessing the study outcomes blind to the patient interventions. The two researchers were kept in separate partitioned areas to minimize communication between them, with the second researcher unaware of which study arm the patient was assigned to. Following the education, patients were asked to keep the pamphlets within sealed envelopes we provided to prevent the GP and the second researcher from seeing the pamphlets. GPs were not told each patient's allocation and were informed not to attempt to find out what topic each patient was counseled on. However, GPs were not blinded to the study aims, and we emphasized that they should educate their patients about antibiotics where appropriate. We assessed the adequacy of blinding for the GPs through intraconsult questionnaires for each patient and found that in 99% of instances, GPs were unaware of which arm the patient was in.

### Outcome measures.

The primary outcome was the proportion of patients in each arm that were prescribed antibiotics. The secondary outcomes were patients' agreement on a 4-point Likert scale to the following three statements: that the education had improved their understanding about causes of URTI, that they were worried about the side effects of antibiotics, and that antibiotics are not needed most of the time for URTI.

### Sample size calculations.

In the absence of data on antibiotic prescription rates for URTI patients in Singapore, we assumed a range of estimates from 10% to 30% on the basis of findings from studies in regional countries with health indices comparable to those of Singapore (8, 29, 30). We estimated that, with equal numbers in the intervention and control arms and assuming that as many as 30% would receive antibiotics in the absence of any intervention, a sample size of about 900 patients would have 80% power to detect the effect of an intervention that reduced the prescription rate by >30% at a *P* value of less than 0.05. Alternatively, if prescription rates were as low as 10%, then the intervention would have to reduce prescription rates by >50% for us to have a similar power to detect its effect.

### Data management and data analysis.

We analyzed the effect of the intervention on antibiotic prescription using odds ratios (ORs) and 95% confidence intervals (CIs) using a multilevel model (with doctor-level groupings) to account for potential clustering of results at the GP level. Stratified analyses were performed to investigate the effect of age, sex, education level, and ethnicity on the efficacy of the intervention and to identify any variations by GP. In addition, univariate and multivariate multilevel analyses were used to investigate patient and doctor-level factors associated with antibiotic prescription. Patient factors included the patient's age, sex, educational background, self-reported ethnicity, and patient beliefs, whereas doctor factors included the sex of the doctor, the years since the doctor qualified as a registered medical practitioner, and whether the doctor was a member of a larger corporate entity, such as a limited-liability partnership or clinic chain. Differences in patients' postconsultation views about URTIs and antibiotics between the control and intervention study arms were analyzed as ordinal outcomes with Mann-Whitney U tests.

All data were analyzed using Stata for Windows, version 11 (Stata Corporation, College Station, Texas, USA). *P* values of less than 0.05 were considered statistically significant.

### Ethics approval.

The institutional review board of NUS approved the study (reference B-14-259).

## Supplementary Material

Supplemental material
