# Supplementary material for: Results from a Patient-Based Health Education Intervention in Reducing Antibiotic Use for Acute Upper Respiratory Tract Infections in the Private Sector Primary Care Setting in Singapore
Source: Antimicrob Agents Chemother. 2017 Apr 24;61(5):e02257-16. doi: 10.1128/AAC.02257-16 (PMC5404603; doi:10.1128/AAC.02257-16)
Supplement: Supplemental material [file supp_61_5_e02257-16__index.html]

Results from a Patient-Based Health Education Intervention in Reducing Antibiotic Use for Acute Upper Respiratory Tract Infections in the Private Sector Primary Care Setting in Singapore — Supplemental material 

# Results from a Patient-Based Health Education Intervention in Reducing Antibiotic Use for Acute Upper Respiratory Tract Infections in the Private Sector Primary Care Setting in Singapore

## Supplemental material

- Supplemental file 1 -

  Supplemental Text S1: education script, intervention and control arms.

  PDF, 506K
