# Supplementary material for: Results from a Patient-Based Health Education Intervention in Reducing Antibiotic Use for Acute Upper Respiratory Tract Infections in the Private Sector Primary Care Setting in Singapore
Source: Antimicrob Agents Chemother. 2017 Apr 24;61(5):e02257-16. doi: 10.1128/AAC.02257-16 (PMC5404603; doi:10.1128/AAC.02257-16)
Supplement: Supplemental material [file AAC.02257-16_zac005176105s1.pdf]

Intervention Arm

STEP 1 : RECRUITMENT OF PATIENT

Hi Sir/Ma'am, my name is ______, and I am a 4th year medical student from the NUS Yong Loo Lin School of Medicine. As part of our curriculum, we are carrying out a research study looking at the prescription of antibiotics for common day-to-day infections and I would like to invite you to help us. We are working under the mentorship of Dr Mark Chen from Saw Swee Hock School of Public Health, NUS.

This research study seeks to find out the public's perception of antibiotics and the family physicians' prescribing behaviours. Through a short survey, we are also hoping to raise awareness and understanding in antibiotic prescription. We would therefore like to invite you to take part in our project. Not to worry, your response would be anonymous.

The doctor who is seeing you is also aware of our project and has given us his approval.

Before we begin, we would ask you a few short questions to see if you are eligible for our study. It would take less than 1 minute.

STEP 2 : ADMINISTER ELIBIGIBILTIY QUESTIONNAIRE Thank

you for answering my questions. It appears that:

SCENARIO 1: You do not fit the inclusion criteria for our study. Thank you so much for your time anyway, and we hope you get well soon.

OR

SCENARIO 2: You fit the inclusion criteria for our study. Please allow us explain some of the details of our research before you decide if you would like to be part of our study.

STEP 3 : EXPLANATION OF THE PATIENT INFORMATION SHEET AND TAKE CONSENT

Ensure that:
- Objective of the project has been explained
- Patient is given the option to opt out at any time
- Patient understands that participation is fully voluntary

Before you see your doctor, we would conduct a short survey that looks at your understanding and past experiences of some of the common infections and the use of antibiotics
After which, we have a simple brochure here to help you understand the common cough and flu. After you see the doctor, we would conduct another short survey with 3 questions and take a look at the medications you have been prescribed, if that's all right with you.

This entire process would not take up more than 10 minutes of your time.

If you are willing, I would like to take your official consent. You can keep this information sheet for your reference.

STEP 4: ADMINISTER PRE-CONSULT KAP QUESTIONNAIRE

STEP 5 : EDUCATION WITH BROCHURE (EITHER CONTROL/INTERVENTION)

Antibiotics script

Sir/Ma'am, from what we gathered just now and which you probably already know, you seem to be having an infection. These infections are common and are usually caused by bacteria or viruses. Knowing what caused your infection is important for you to make a more informed decision on the treatment you may require.

- ✐ **Causes of common cough and cold**

Most of the infections, up to 75%, that people experience, like your everyday cough, cold and runny nose are caused by viruses (75%). Many people may feel that only antibiotics can relieve their symptoms. However, it is IMPORTANT to understand that these antibiotics will NOT cure you of your viral infections.

- ✐ **Symptoms of illness that require antibiotics**

We often think that yellow mucous indicates a need for antibiotics, but this is not always the case. We only need antibiotics when the infection is caused by bacteria, and there are many clues which your doctor may use to determine the likelihood of you having a bacterial infection. These include the duration of your illness, your past medical illnesses, if you have recently had contact with any sick people, and many more.

- ✐ **What are antibiotics for**

Antibiotics are only effective in treating illnesses caused by bacteria, which are less common. Thus, it's important that you don't take antibiotics if you do not have a bacterial infection.

- ✐ **Side effect of unnecessary antibiotics**

<u>Allergic reaction</u>: Taking antibiotics unnecessarily can cause side effects as mild as stomach discomfort and as severe as allergic reactions which may result in hospitalization.

<u>Colonization of good gut flora</u>: Can 'kill off' the good bacteria in our gut and do us more harm than good. (elaborate more on the concept of good bacteria that lives on us)

<u>Resistance</u>: By inappropriately using antibiotics, bacteria will be able to develop resistance to the antibiotics and will be harder to kill over time.

<u>Seriousness of the issue</u>: This is a serious issue and the hospitals are facing difficulties in treating super-bugs because we are breeding these super bugs faster than the development of new antibiotics.

<u>Others</u>: Can also save money from not purchasing medication that you do not really need. We do not wish for the public to take medication unnecessarily, resulting in more harm than good.

<u>Emphasize role of doctor</u>: If your doctor does not prescribe you antibiotics, it is because he has carefully assessed you and does not think you currently have a bacterial infection. However, at times, a secondary bacterial infection occurs concurrently and if your symptoms worsen, it is advisable for you to revisit the doctor.

<u>Good antibiotic usage habits</u>: When the doctor does prescribe you antibiotics, it is very important that you finish the course of the antibiotics to ensure that the medicine kills off all the bacteria. Otherwise, the next time you are sick, because of antibiotic resistance, you might be infected with stronger bacteria that

is more difficult to kill and causes a more serious illness. It actually does more harm than good if you take less than prescribed antibiotics.

Answer any questions that the patient might have

Control Arm

STEP 1 : RECRUITMENT OF PATIENT

Hi Sir/Ma'am, my name is _____, and I am a 4th year medical student from the NUS Yong Loo Lin School of Medicine. As part of our curriculum, we are carrying out a research study looking at the prescription of antibiotics for common day-to-day infections and I would like to invite you to help us. We are working under the mentorship of Dr Mark Chen from Saw Swee Hock School of Public Health, NUS.

This research study seeks to find out the public's perception of antibiotics and the family physicians' prescribing behaviours. Through a short survey, we are also hoping to raise awareness and understanding in antibiotic prescription. We would therefore like to invite you to take part in our project. Not to worry, your response would be anonymous.

The doctor who is seeing you is also aware of our project and has given us his approval.

Before we begin, we would ask you a few short questions to see if you are eligible for our study. It would take less than 1 minute.

*****USE THE SIMPLE SURVEY QUESTIONS TO PICK UP ON WHO IS APPROPRIATE FOR STUDY FIRST*****

STEP 2 : ADMINISTER ELIBIGIBILTIY QUESTIONNAIRE Thank

you for answering my questions. It appears that:

SCENARIO 1: You do not fit the inclusion criteria for our study. Thank you so much for your time anyway, and we hope you get well soon.

OR

SCENARIO 2: You fit the inclusion criteria for our study. Please allow us explain some of the details of our research before you decide if you would like to be part of our study.

STEP 3 : EXPLANATION OF THE PATIENT INFORMATION SHEET AND TAKE CONSENT

Ensure that:

- Objective of the project has been explained
- Patient is given the option to opt out at any time
- Patient understands that participation is fully voluntary

Before you see your doctor, we would conduct a short survey that looks at your understanding and past experiences of some of the common infections and the use of antibiotics
After which, we have a simple brochure here to help you understand the common cough and flu. After you see the doctor, we would conduct another short survey with 3 questions and take a look at the medications you have been prescribed, if that's all right with you.

This entire process would not take up more than 10 minutes of your time.

If you are willing, I would like to take your official consent. You can keep this information sheet for your reference.

STEP 4: ADMINISTER PRE-CONSULT KAP QUESTIONNAIRE

Control Arm

STEP 5 : EDUCATION WITH BROCHURE (CONTROL)

Control Arm

Vaccine Script

Sir/Ma'am, from what we gathered just now and which you probably already know,you seem to be having an infection. These infections are common and are usually caused by bacteria or viruses.

Firstly, we have to understand that even though most of these infections are mild and go away spontaneously, there is this virus called the influenza virus and it can affect 10-15% of Singapore's population. It can cause severe hospitalization, and even deaths.

Sir/Mdm, do you get regular yearly flu vaccinations? Or do you know that such a vaccine exists?

[Patient will answer yes or no]

If the patient answers **YES**:

[You can still educate the same way, perhaps asking the participant more questions and clarifying any misconceptions they may have. You can perhaps also ask them to encourage vaccination to their family members and ensure that the participant knows that the vaccination needs to be renewed yearly. If they know about the vaccine but do not go for it, you can ask them why and educate accordingly]

If patient answers **NO:**

Okay! So now, perhaps I can tell you more about flu viruses and the vaccine available that can protect you from being infected with these viruses.

In the brochure (point to the segment) there is a list of common symptoms that people with the flu may commonly present with.

So you may be wondering how do these flu viruses spread? These viruses are present in the droplets produced when a person with the flu coughs, sneezes or talks. These droplets can spread to people or surfaces within 1 to 3 metres and if you are in direct contact with these droplets or happen to touch the contaminated surfaces and subsequently touch your eyes, nose or mouth, you put yourself at risk of getting infected as well!

You can protect yourself from getting these infections by practicing good hand hygiene but that does not protect you from being infected by direct contact with the droplets. So another way in which you can protect yourself would be by getting vaccinated. Though getting the vaccine may involvegetting an injection, the pain you may feel is only very temporary. On the other hand, there are many benefits. The vaccine will protect you from the influenza virus and prevent you from getting severe illness. Moreover, protecting yourself would also help protect your loved ones as well!

The vaccination would be the most effective if administered before the flu season starts. In Singapore, the flu season is usuallybetween December to January & May to July. The vaccine contains the 3 most common strains of viruses currently circulating

and has to be renewed yearly as the strains constantly change, and your body's immunity to the virus may decline over time.

Though it is recommended for all healthy individual to be vaccinated, there are certain groups of people to whom the vaccine is more highly recommended. These are the people who are at high risk of getting severe flu infections. The main groups of people are those who are 65 years or older and children 6 months to 5 years, people with weakened immunity or with chronic lung, heart or metabolic disorders.

The flu vaccine is very safe as the virus component contained in the vaccine is inactivated; therefore, women who are pregnant can also able be vaccinated. However, you should take note that the vaccine is contraindicated in people with severe allergic reaction to previous flu vaccinations or have an egg allergy. If you are unsure if you have any medical condition or allergies that may be a contraindication to the vaccine, you should approach your GP or a go to a polyclinic to ask about it.

So if you're considering going for a vaccination, you can easily get it from any nearby hospitals, polyclinics and GP clinics. Payment for these vaccinations can be made via your Medisave account if you have a higher risk of developing influenzarelated complications. As mentioned before, the vaccine is generally safe but you may experience minor side effects like body aches and swelling/pain/tenderness at the injection site, which may last 1-2 days after the injection.

I hope that you have learned much about the spread of the flu virus and the vaccine available to help you prevent it. Though it may be easy to underestimate the Flu virus, it's important to understand that it could potentially lead to severe illnesses too. Thus, practicing simple hand hygiene methods, and getting easily available vaccinations will go a long way into preventing the complications of the Flu virus and maintaining a healthy life. Thank you for your time Sir/Ma'am.

Do you have any questions for me?

If the patient answers **YES**:

[You clarify any doubts that they may have]

If patient answers **NO:**

Okay Sir/Ma'am, we would like you to keep this brochure for your future reference. To aid us in our study, we would also like to request that you not reveal the brochure or the contents of the education you received from us to your doctor during your consultation. We would also like to hand you a slip of paper for you to pass to the doctor. There is some information the doctor will fill in, following which he/she will pass this paper back to you after the consult. We will be waiting outside to collect this slip of paper from you after.

Any last questions for me?

If patient answers **NO:**

Control Arm

Thank you once again for your time and co-operation in participating in our study. You can proceed to the consultation with your doctor.

Answer any questions that the patient might have
